# On the Influence of Poisons upon Animal Heat as the Cause of Death

**Published:** 1852-11

**Authors:** E. Brown Séquard

**Affiliations:** Paris


*On the influence of Poisons upon Animal Heat as a Cause of Death.* By E. Brown Séquard, M. D., of Paris. Prévost and Chossat, and after them, M. Magendie, have ascertained that death occurs quickly in mammals when their temperature is notably diminished. My experiments confirm the correctness of that statement. The diminution of animal heat in mammals is so dangerous, that in one case I have seen death take place in a rabbit after a diminution of only 22° F. (12° Cs.) I have never observed any animal continuing to live when I had diminished its temperature more than 44° F. (24°.5 Cs.) I have found the law established by Chossat perfectly correct, according to which the diminution of animal heat necessary for killing is less and less, in proportion to the rapidity with which that diminution takes place.

It is very probable that in all the cases where, in consequence either of disease, or of a wound, or of poison, the temperature of man is diminished many degrees, his life is in danger from the very fact of that diminution. It is thus in cholera, in sclerema, in certain cases of palsy, in cases of great disturbance of the respiration, in fractures and luxations of the vertebral column, in the cervical, and even in the dorsal regions, in cases of profuse hæmorrhage, and in many cases of poisoning when death is not rapidly produced.

It has been long known that temperature is diminished in poisoned persons; and there are but few cases of poisoning on record in which it is not said that the patient was cold. Chossat has found that a dog, into whose veins he had injected opium, had its temperature diminished from 105° to 62.6° F. (40°.3 to 17° Cs.,) 22 hours after the injection. Brodie has found that many poisons act upon animal heat so as to diminish it considerably. Demarquay and Dumeril, junior, and later, these two experimenters, joined with Lecointre, have found the same thing as Brodie in many toxic agents. I have made very numerous experiments on this subject, and some of their results have been published before the last papers of Demarquay, Dumeril and Lecointre.*

I have stated that many poisons, either injected in the veins or absorbed by the vessels of the skin or of the digestive canal, may diminish sufficiently the temperature of Guinea pigs and rabbits to produce death. This occurs when the dose of the poison is not large enough to kill in less than four or five hours. These poisons may kill only by their action upon animal heat. It may be so with opium, cyanhydric acid, the cyanide of mercury, hyoscyamus, digitalis, belladonna, tobacco, euphorbia, camphor, alcohol, acetic, oxalic, sulphuric, azotic, chlorohydric acids much diluted, and some oxalates.

Of course the action of these poisons is the greater, the colder the atmosphere; but it is not always immediately so, and instead of diminishing the animal heat, many may increase it for a time, especially when the temperature of the atmosphere is elevated.

I have discovered that a dose of one of these poisons, sufficient to kill an animal, when there is no obstacle to the diminution of its temperature, may be unable to destroy life when the temperature of the animal is main-

* See Gaz. Med. de Paris, 1849, t. iv., p. 644.

tained by artificial means to its normal degree, or not far from it. My experiments have been conducted as follows: —

Equal doses of poison were given, simultaneously, to two animals, as much alike one another as possible. One of them was left in a room at a temperature of from 46 to 50° F., (8 to 10° Cs.,) and the other was kept not far from a chimney, in a place where the air was at from 75 to 86° F., (24 to 30° Cs.) The first was dead after a certain number of hours, or sometimes one or two days, having its temperature much diminished. The other, on the contrary, had no perceptible diminution of its temperature, and was generally cured very soon. Therefore, when taken in certain doses, many poisons may kill only by their influence on animal heat, physicians, in cases of poisoning, should try as much to prevent the diminution of temperature, as to expel the poison or to act against it by an antidote, by pulmonary insufflation, or otherwise.

In experiments which I have made lately on the action of very pure digitaline, that had been prepared by M. Quévenne, the celebrated chemist, who has made such interesting and accurate researches on digitalis and the substances of which it is composed, I have found that this poison may also diminish temperature. I believe it is easy to explain the contradiction existing between Traube and Stannius, as regards the influence of digitalis on animal heat. When the atmosphere, in which the animal is, is cold, then its temperature may be diminished by digitalis or digitaline, but when it is warm the diminution does not take place, or it is very small. But, of course, if the dose is sufficient to kill very quickly, then it is indifferent whether the atmosphere is cold or not, because there may be not time enough for the dimin ution of the temperature of the animal.

I have to relate another fact which, I believe, ought to be considered as analogous to the preceding. It is that kind of poisoning which occurs when a layer of oil, of varnish or of gelatin, put on the skin of a warm-blooded animal. Death, then, is very probably produced by a substance unknown until now, and which is secreted by the skin. The layer of oil, varnish, or gelatin preventing that secretion taking place, that unknown substance becomes accumulated in the blood, and then are produced the phenomena so well studied by MM. Fourcault, Becquerel, Breschet, and Magendie. I have found that in such a case the animals may live, if the atmosphere in which they are kept is at a temperature inferior to 79 or 80° F., (26 or 27° Cs.) In these circumstances their temperature is not sensibly diminished, while it diminishes much when the atmosphere is cold. Therefore it is especially by their loss of warmth that animals are killed, when their body has been entirely covered with oil, varnish, or gelatin.—*Ib.*

